# The impact of previous endoscopic treatments on functional outcome after cricotracheal resection

**DOI:** 10.1093/ejcts/ezae105

**Published:** 2024-05-18

**Authors:** Matthias Evermann, Thomas Schweiger, Veronika Kranebitter, Imme Roesner, Clemens Aigner, Doris-Maria Denk-Linnert, Konrad Hoetzenecker

**Affiliations:** Department of Thoracic Surgery, Medical University of Vienna, Vienna, Austria; Department of Thoracic Surgery, Medical University of Vienna, Vienna, Austria; Division of Phoniatrics and Speech Language Therapy, Department of Otorhinolaryngology, Medical University of Vienna, Vienna, Austria; Division of Phoniatrics and Speech Language Therapy, Department of Otorhinolaryngology, Medical University of Vienna, Vienna, Austria; Department of Thoracic Surgery, Medical University of Vienna, Vienna, Austria; Division of Phoniatrics and Speech Language Therapy, Department of Otorhinolaryngology, Medical University of Vienna, Vienna, Austria; Department of Thoracic Surgery, Medical University of Vienna, Vienna, Austria

**Keywords:** Subglottic stenosis, Cricotracheal resection, Endoscopic treatment, Functional outcome

## Abstract

**OBJECTIVES:**

Treatment options for benign subglottic stenosis include endoscopic techniques or open surgery. Although endoscopic treatment is less invasive, a considerable proportion of patients develop recurrent stenosis. Endoscopic pretreatments do not exclude patients from a later surgical repair; however, the impact of previous endoscopic treatment attempts on functional outcome after open surgery is unknown.

**METHODS:**

All patients, who received a cricotracheal resection (CTR) between January 2017 and June 2023 at the Department of Thoracic Surgery, Medical University of Vienna, were included in this retrospective study. Patient characteristics, surgical variables and postoperative outcome including a detailed functional assessment were analysed.

**RESULTS:**

A total of 65 patients received a CTR during the study period, of which 40 were treatment naïve and 25 had a median of 2 (range 1–9) endoscopic pretreatments. Less-invasive voice-sparing CTR or standard CTR were more often possible in treatment-naïve patients. In contrary, pretreated patients regularly required extended procedures (*P* = 0.049). Three or more endoscopic treatments resulted in a significantly lower mean fundamental frequency (F0) after open repair (*P* = 0.048). In addition, a trend towards smaller mean sound pressure levels, a higher voice handicap index, higher impairments in RBH scores (roughness, breathing and hoarseness) and a higher dysphagia severity index was found in pretreated patients. The respiratory outcome after surgery was comparable between both groups.

**CONCLUSIONS:**

Multiple endoscopic pretreatments lead to worse voice quality after CTR. The impact of prior endoscopic treatment before surgical repair should be considered when discussing treatment options with patients suffering from subglottic stenosis.

## Graphical Abstract

**Figure:**
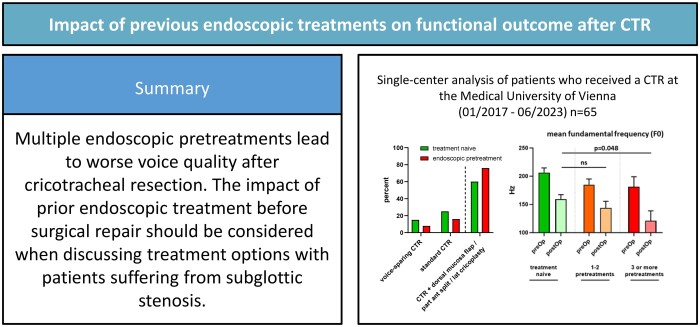

## INTRODUCTION

Benign stenoses of the subglottic airway are rare and their aetiology is heterogeneous. The most common causes of subglottic stenosis are idiopathic (idiopathic subglottic stenosis) or scarring of the airway after a tracheostomy or a traumatic intubation [1]. The optimal treatment for subglottic stenosis is still a matter of debate. Endoscopic treatment options include balloon dilatation, dilatation with rigid scopes or cautery/laser enlargements [2–5]. Although endoscopic options lead to an immediate relief of symptoms, they result in restenosis in a high percentage of cases. Cricotracheal resection (CTR) is superior to endoscopic treatment options, as it offers an extremely low recurrence rate [6, 7]. However, CTR is offered only by a limited number of thoracic surgical departments, as it is considered technically challenging and requires more infrastructure compared to endoscopic treatments. In addition, CTR and especially extended CTR has a mild to moderate effect on postoperative voice quality as it usually reduces the voice pitch and voice range of patients.

It has previously been shown that CTR is feasible in patients, who had undergone (multiple) previous endoscopic treatments [8–10]. However, none of the published series investigated the impact of pretreatments on laryngeal functions (voice quality, swallowing) following a definitive surgical repair.

## PATIENTS AND METHODS

### Ethics statement

This study was approved by the Ethics Committee of the Medical University of Vienna (EK# 1639/2023). Due to the retrospective nature of the study, patient consent was waived.

### Study population

All patients with benign subglottic stenosis, who received a CTR between January 2017 and June 2023 at the Medical University of Vienna, Austria, were included in this retrospective single-centre analysis (*n* = 65). Patients were grouped into (i) treatment-naïve patients (*n* = 40) or (ii) patients who had undergone endoscopic pretreatment(s) (*n* = 25). Paediatric patients, patients with a previous (failed) laryngotracheal resection and patients who required a laryngotracheal reconstruction with cartilage graft enlargements [11, 12] were excluded. Medical records were analysed to define patient characteristics, clinical variables, anamnestic factors, functional and endoscopic measurements, surgical interventions and long-term outcomes.

### Surgical techniques

In general, the aim of surgical repair was to completely remove all diseased parts of the airway followed by an end-to-end anastomosis using healthy mucosa. After dissecting the cervical airway, the trachea was opened below the stenosis. Using a step-wise approach, resection lines were moved cephalad until clear resection margins were reached. A standard CTR included a resection of the cricoid arch followed by a thyreo-tracheal anastomosis, as described elsewhere [13]. If the stricture involved the posterior subglottic airway, a dorsal mucosectomy was added. In cases with extensive narrowing, a lateral cricoplasty [14] or a partial anterior laryngeal split [15] became necessary to obtain a sufficient airway lumen. These extended procedures were opposed by voice-sparing methods, where parts of the cricoid arch and the cricothyroid joint could be preserved [16]. In case of clinically relevant swelling of the vocal fold at the end of the procedure, a utility tracheostomy (size #5) was inserted 3–4 rings below the anastomosis. Procedures were grouped based on their complexity: (i) voice-sparing CTR, (ii) standard CTR and (iii) CTR with dorsal mucosal flap and/or partial anterior split and/or lateral cricoplasty.

### Functional evaluation

All patients received vocal audio recordings and an extensive endoscopic and functional evaluation of voice and swallowing before surgery as well as 3 months postoperatively.

#### Voice

Voice assessment was performed according to the recommendations of the European Laryngological Society (ELS) by a dedicated team of Phoniatricians and Speech Language Pathologists [17, 18]. Voice assessment included transnasal videolaryngostroboscopy with chip-on-the tip videoendoscopes for evaluation of respiratory and phonatory mobility of the vocal folds, glottic closure and the level of phonation (glottic or supraglottic). Moreover, perceptive evaluation according to the RBH grading [roughness, breathing and hoarseness of the voice ranging from 0 (normal) to 3 (severe impairment) [19] and the 9-point Voice Handicap Index were documented [20]]. The voice range profile (phonetogram) measured the mean fundamental frequency F0 (in Hz), voice range (in semitones) and sound pressure level of fundamental frequency (in dB; DiVAS software, XION GmbH, Berlin, Germany). Spirometry was used to evaluate respiratory function.

#### Swallowing

For evaluation of swallowing, a flexible endoscopic evaluation of swallowing (FEES) with digital recording was conducted based on to the Langmore Protocol [21], using standardized bolus volumes and consistencies in accordance to the International Dysphagia Diet Standardisation Initiative (The International Dysphagia Diet Standardisation Initiative 2019; https://iddsi.org/framework/Licensed under the Creative Commons Attribution Sharealike 4.0) for thin, slightly thick, moderately thick liquids and solid consistencies (easy to chew and regular) were conducted. The Penetration–Aspiration Scale was used for classification of swallowing function [22]. In addition, patients were asked to self-assess their swallowing function using a structured form [1 (no impairment) to 7 (severe dysphagia); dysphagia severity index] [23]. All patients received a bronchoscopy in general anaesthesia before the surgery to measure the extent and length stenosis, distance to vocal fold level and total length of the trachea. Three months after the surgery, another bronchoscopy was performed to assess the healing of the anastomosis.

### Statistical analysis

Statistical analysis was performed using GraphPad Prism 10 (GraphPad Software Inc., California, USA). Due to the small sample sizes, Mann–Whitney *U*-tests were used to compare differences between 2 independent groups of continuous variables. Wilcoxon matched-pairs signed rank test to compare dependent variables. A chi-square test was used to compare the categorical variables of different resection techniques. Values were expressed with mean ± error of the mean. Categorical variables were presented as values and percentages. All tests were two-sided unless otherwise noted. *P*-values <0.05 were considered as statistically significant.

## RESULTS

### Patient demographics

The study population mainly consisted of female patients (88%). The most common diagnosis was idiopathic subglottic stenosis (66%) followed by tracheostomy-associated stenosis (12%) and post-intubation stenosis (11%). The vast majority of patients were referred with a Myer-Cotton grade 3 stenosis (70–99% lumen reduction). Patients in the pretreatment group had a median of 2 prior endoscopic treatments (range 1–9) before they were referred for surgical repair. Treatment-naïve patients and patients with previous endoscopic pretreatments did not differ in regard to age, gender, underlying diagnosis, Myer-Cotton grade, length of stenosis and comorbidities (Table 1).

**Table 1:** Patient characteristics

	Total (*n* = 65)	Treatment naive (*n* = 40)	Endoscopic pretreatment (*n* = 25)
Age (years), median (IQR)	51 (44–62)	54 (41–62)	50 (45–57)
Gender (m %/f %)	12%/88%	15%/85%	8%/92%
Diagnosis, *n* (%)			
iSGS	43 (66%)	27 (68%)	16 (64%)
Tracheostomy-associated stenosis	8 (12%)	6 (15%)	2 (8%)
Postintubation stenosis	7 (11%)	4 (10%)	3 (12%)
GPA	6 (9%)	2 (5%)	4 (16%)
Others	1 (2%)	1 (2%)	0 (0%)
Myer-Cotton grade, *n* (%)			
Grade 2	6 (9%)	6 (15%)	0 (0%)
Grade 3	59 (91%)	34 (85%)	25 (100%)
Length of stenosis (cm), mean (95% CI)	2.1 (1.3–2.8)	2.1 (1.3–2.8)	2.1 (1.4–2.9)
Comorbidities			
Hypo/hyperthyroidism	16	12	4
Arterial hypertension	10	7	3
Diabetes	2	2	0
COPD/chronic lung disease	4	4	0
GERD	5	1	4
Chronic kidney disease	1	1	0
Others			
Number of endoscopic pretreatments, median (range)	0	0	2 (1–9)

COPD: chronic obstructive pulmonary disease; IQR: interquartile range; iSGS: idiopathic subglottic stenosis.

### Procedural details

**Figure 1:**
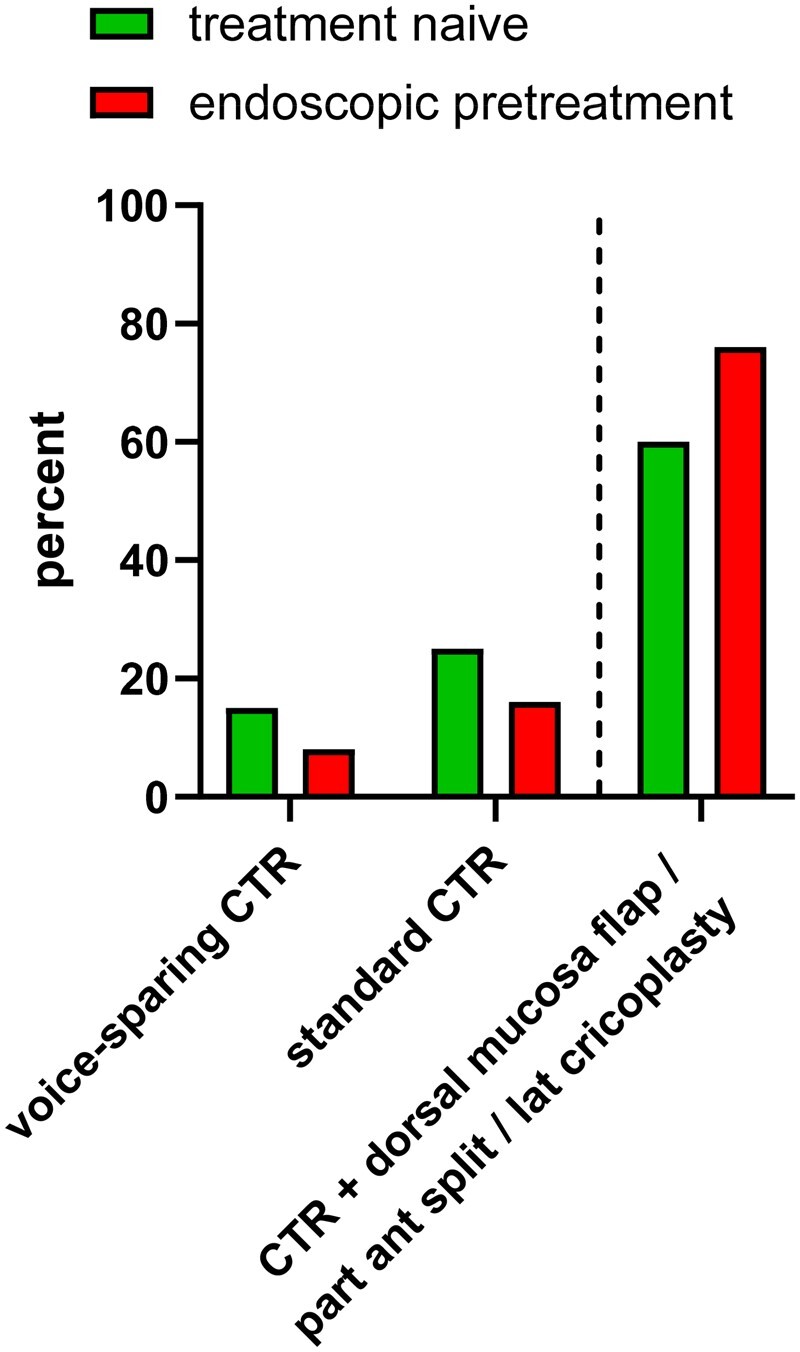
Extent of resection in treatment-naïve and endoscopically pretreated patients. Pretreated patients regularly required extended procedures (*P* = 0.050). CTR: cricotracheal resection.

The complexity of resection techniques was significantly higher in pretreated patients (*P* = 0.049). Voice-sparing CTR could be performed in 6/40 (15%) of treatment-naïve patients but only in 2/25 (8%) of patients with prior endoscopic treatment attempts. In addition, a lateral cricoplasty was necessary more often in pretreated compared to treatment-naïve patients (24% vs 17%; Fig. 1). We did not capture any difference in regard to resection length, odds ratio time, the need for postoperative utility tracheostomy or complication rates between the 2 study groups (Table 2). In general, complication rates were low. Postoperative vocal fold oedema (*n* = 4) and transient soft tissue emphysema (*n* = 2) were the most common complications. Of note, none of the patient developed a necrosis or dehiscence of the anastomosis. Patients were usually transferred to the intensive care unit for 1 night for surveillance and had a median hospital stay of 6 days. With a mean follow-up of 55.1 months, none of the patients required a reintervention or developed a restenosis.

**Table 2:** Procedural details

	Total (*n* = 65)	Treatment naive (*n* = 40)	Endoscopic pretreatment (*n* = 25)
Type of resection, *n* (%)			
Voice sparing CTR	8 (12)	6 (15)	2 (8)
CTR	14 (22)	10 (25)	4 (16)
CTR + dorsal mucosal flap	29 (44)	16 (40)	13 (52)
CTR + part ant split + dorsal mucosal flap	1 (2)	1 (3)	0 (0)
CTR + lateral cricoplasty + dorsal mucosal flap	13 (20)	7 (17)	6 (24)
Resection length (cm), median (IQR)	2.5 (1.9–3.1)	2.5 (1.8–3.2)	2.5 (1.9–3.1)
OR time (min), mean (95% CI)	137 (97–177)	135 (95–175)	142 (100–184)
Complications, *n* (%)			
Vocal fold oedema	4 (6)	2 (5)	2 (8)
Soft tissue emphysema	2 (3)	1 (3)	–
PostOp utility tracheostomy, *n* (%)	6 (9)	4 (10)	2 (8)
Time until decannulation (days), median	3	4	3
Start of oral intake (POD), median (IQR)	1 (1–2)	1 (1–2)	1 (1–2)
ICU, *n* (%)	47 (72)	30 (75)	17 (68)
ICU length of stay (days), median (range)	1 (1–7)	1 (1–7)	1 (1–2)
Hospital length of stay (days), median (range)	6 (3–16)	6 (3–16)	6 (4–13)
Follow-up (months), mean	55.1	53.3	57.8
Restenosis/reintervention	0%	0%	0%

CTR: cricotracheal resection; ICU: intensive care unit; IQR: interquartile range; OR: odds ratio; POD: postoperative day.

### Functional outcome

**Figure 2:**
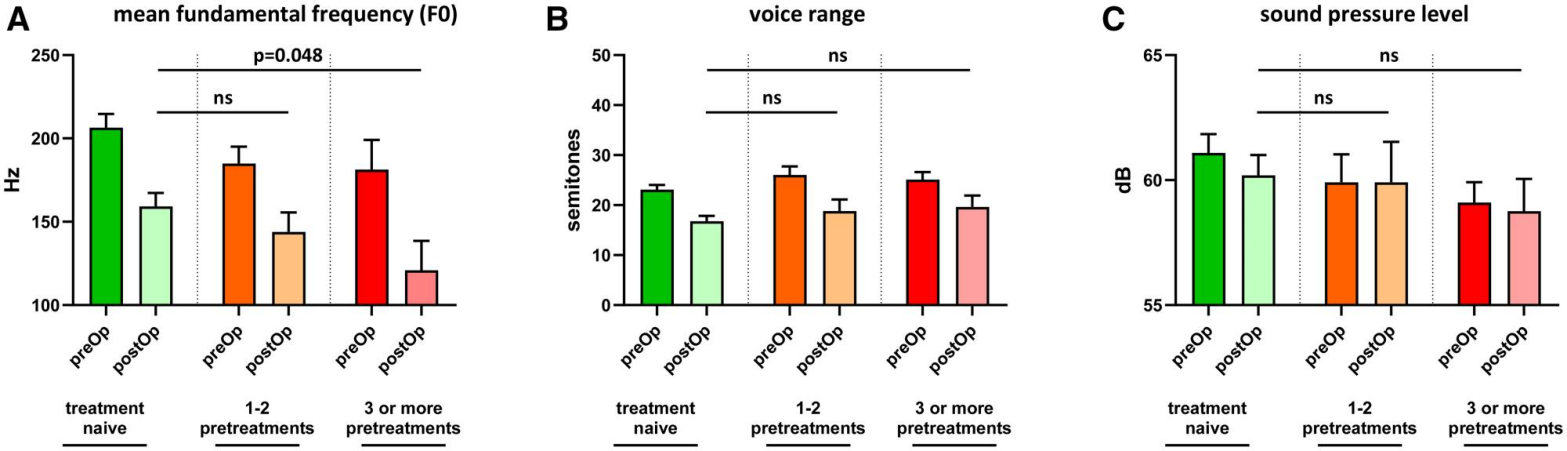
The impact of cricotracheal resection on quantitative measurements of voice quality. (**A**) Multiple endoscopic pretreatments resulted in a significantly lower mean fundamental frequency (*P* = 0.048). Voice range remained unchanged (**B**); however, we observed a trend towards lower sound pressure levels (speaking voice) in pretreated patients after open surgery (**C**).

**Figure 3:**
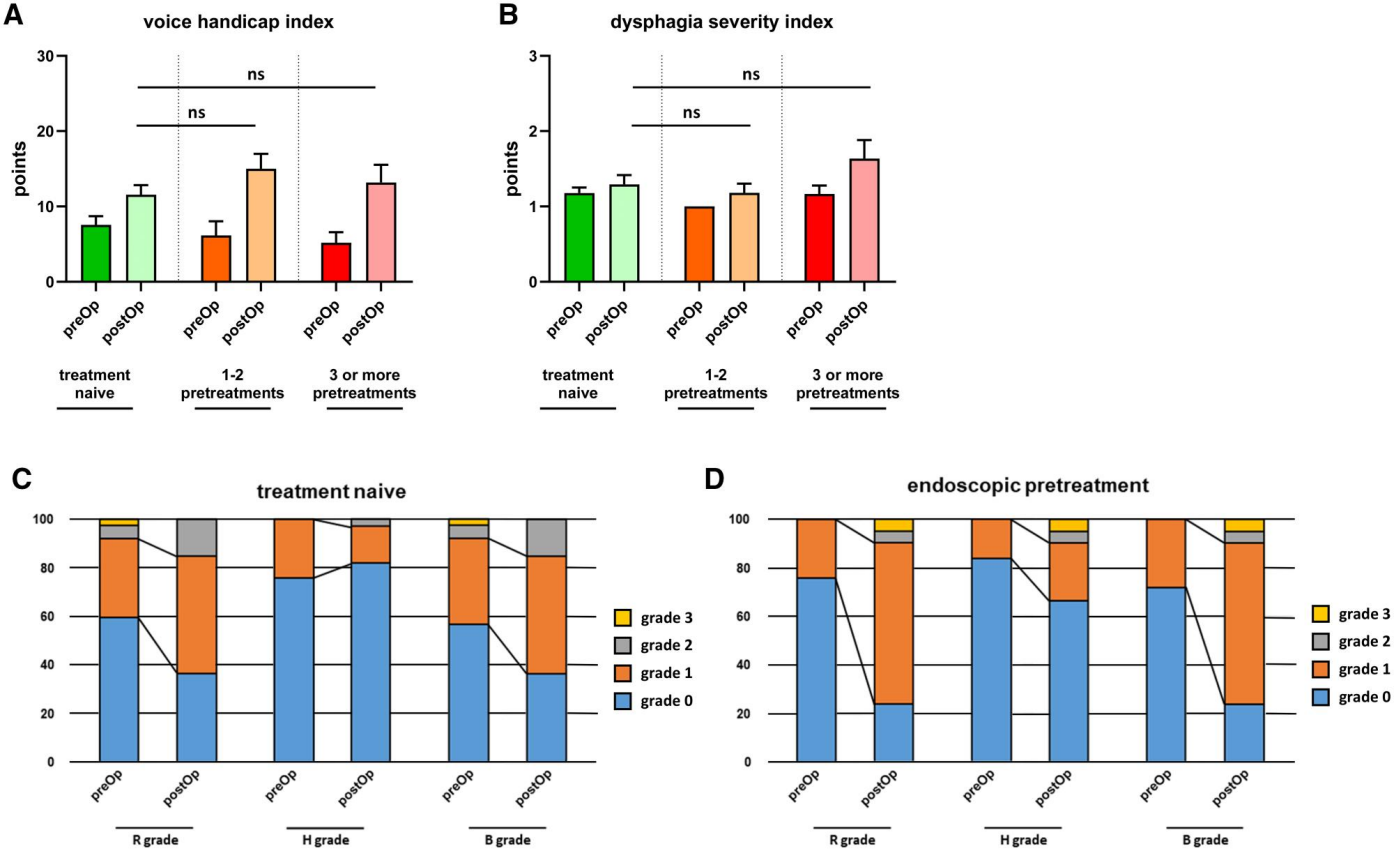
Patient-reported functional outcomes after cricotracheal resections is depicted in (**A**) and (**B**). A trend towards worse voice handicap index and dysphagia severity index was observed. Endoscopically pretreated patients also had a worse RBH grading after cricotracheal resection compared to treatment naïve patients (**C** and **D**).

Functional outcome was measured during a routine follow-up visit 3 months after the operation (Table 3). Postoperative phonation occurred at the level of the vocal folds in all patients (100%). One patient in the treatment-naïve group and 2 patients in the pretreatment group had reduced vocal fold abduction without any evidence of recurrent laryngeal nerve palsy. All patients had a relevant reduction in mean fundamental frequency (F0) and voice range as compared to their preoperative values. Interestingly, the reduction in fundamental frequency was more profound in pretreated patients (Fig. 2; *P* = 0.048). This corresponded to worse subjective outcome in the 9-item voice handicap index, which is describes the patient’s perception of voice quality (Fig. 3A). Evaluation of the RBH score also showed a slightly worse outcome in roughness, breathiness and hoarseness of pretreated patients as compared to treatment-naïve patients with less patients in R grade 0, H grade 0 and B grade 0 (Fig. 3C and D).

**Table 3:** Functional outcome

	Total (*n* = 65)	Treatment naive (*n* = 40)	Endoscopic pretreatment (*n* = 25)
Phonation, *n* (%)			
Preoperative level of phonation			
Vocal cords	65 (100)	40 (100)	25 (100)
Vestibular cords	0 (0)	0 (0)	0 (0)
Postoperative level of phonation			
Vocal cords	65 (100)	40 (100)	25 (100)
Vestibular cords	0 (0)	0 (0)	0 (0)
Preoperative vocal cord movement			
Unremarkable	60 (92.3)	37 (92.5)	23 (92)
Reduced	5 (7.7)	3 (7.5)	2 (8)
Immobile	0 (0)	0 (0)	0 (0)
Postoperative vocal cord movement			
Unremarkable	64 (98)	40 (100)	24 (96)
Reduced	1 (2)	0 (0)	1 (4)
Immobile	0 (0)	0 (0)	0 (0)
Preoperative glottic closure			
Complete	64 (98)	40 (100)	24 (96)
Incomplete	1 (2)	0 (0)	1 (4)
Postoperative glottic closure			
Complete	63 (97)	38 (95)	25 (100)
Incomplete	2 (3)	2 (5)	0 (0)
RBH score			
Preoperative Roughness			
Grade 0	41 (63.1)	22 (55)	19 (76)
Grade 1	18 (27.7)	12 (30)	6 (24)
Grade 2	2 (3.1)	2 (5)	0 (0)
Grade 3	1 (1.5)	1 (2.5)	0 (0)
ND	3 (4.6)	3 (7.5)	0 (0)
Preoperative breathiness			
Grade 0	49 (75.4)	28 (70)	21 (84)
Grade 1	13 (20)	9 (22.5)	4 (16)
Grade 2	0 (0)	0 (0)	0 (0)
Grade 3	0 (0)	0 (0)	0 (0)
ND	3 (4.6)	3 (7.5)	0 (0)
Preoperative hoarseness			
Grade 0	39 (60)	21 (52.5)	18 (72)
Grade 1	20 (30.8)	13 (32.5)	7 (28)
Grade 2	2 (3.1)	2 (5)	0 (0)
Grade 3	1 (1.5)	1 (2.5)	0 (0)
ND	3 (4.6)	3 (7.5)	0 (0)
Postoperative roughness			
Grade 0	17 (26.2)	12 (30)	5 (20)
Grade 1	30 (46.2)	16 (40)	14 (56)
Grade 2	6 (9.2)	5 (12.5)	1 (4)
Grade 3	1 (1.5)	0 (0)	1 (4)
ND	11 (16.9)	7 (17.5)	4 (16)
Postoperative breathiness			
Grade 0	41 (63.1)	27 (67.5)	14 (56)
Grade 1	10 (15.4)	5 (12.5)	5 (20)
Grade 2	2 (3.1)	1 (2.5)	1 (4)
Grade 3	1 (1.5)	0 (0)	1 (4)
ND	11 (16.9)	7 (17.5)	4 (16)
Postoperative hoarseness			
Grade 0	17 (26.2)	12 (30)	5 (20)
Grade 1	30 (46.2)	16 (40)	14 (56)
Grade 2	6 (9.2)	5 (12.5)	1 (4)
Grade 3	1 (1.5)	0 (0)	1 (4)
ND	11 (16.9)	7 (17.5)	4 (16)
Voice range (semitones), mean ± SD			
Preoperative	24.1 ± 5.3	23.1 ± 5.3	25.6 ± 5.0
Postoperative	17.7 ± 6.4	16.8 ± 6.1	19.2 ± 6.7
Voice pitch (Hz; mean ± SD)			
Preoperative	200 ± 41	206 ± 47	191 ± 29
Postoperative	158 ± 49	159 ± 45	156 ± 56
Volume level (dB; mean ± SD)			
Preoperative	60.5 ± 3.9	61.1 ± 4.2	59.5 ± 3.1
Postoperative	59.9 ± 4.6	60.2 ± 4.6	59.4 ± 4.5
9-Voice-Handicap-Index (mean ± SD)			
Preoperative	6.83 ± 6.30	7.57 ± 6.69	5.70 ± 5.46
Postoperative	12.5 ± 7.0	11.6 ± 7.1	14.1 ± 6.6
Phonation time (s), mean ± SD			
Preoperative	16.6 ± 6.1	16.7 ± 6.4	16.4 ± 5.4
Postoperative	19.6 ± 7.0	18.7 ± 6.6	21.1 ± 7.4
Swallowing, *n* (%)			
Preoperative swallowing			
Unremarkable	64 (98)	39 (97.5)	25 (100)
Aspiration	0 (0)	0 (0)	0 (0)
Penetration	0 (0)	0 (0)	0 (0)
ND	1 (2)	1 (2.5)	0 (0)
Postoperative swallowing			
Unremarkable	54 (83)	33 (82.5)	21 (84)
Aspiration	0 (0)	0 (0)	0 (0)
Penetration	1 (2)	1 (2.5)	0 (0)
ND	10 (15)	6 (15)	4 (16)
Dysphagia severity index (mean ± SD)			
Preoperative	2.00 ± 4.98	1.51 ± 5.39	2.71 ± 4.23
Postoperative	–0.32 ± 7.80	–1.63 ± 9.54	1.75 ± 2.50
Dysphagia self rating 1–7 (mean ± SD)			
Preoperative	1.14 ± 0.39	1.18 ± 0.45	1.08 ± 0.27
Postoperative	1.34 ± 0.69	1.29 ± 0.71	1.41 ± 0.65
Spirometry			
Peak expiratory flow (%; mean ± SD)			
Preoperative	50.4 ± 16.2	51.8 ± 14.9	48.4 ± 17.9
Postoperative	90.7 ± 18.9	90.2 ± 20.2	91.5 ± 16.7
Forced expiratory volume in 1 s (%; mean ± SD)			
Preoperative	85.5 ± 18.5	87.0 ± 17.9	83.3 ± 19.2
Postoperative	93.9 ± 13.5	94.3 ± 13.5	93.4 ± 13.3

SD: standard deviation.

Although patients in both groups could start oral intake between POD 1 and 2, pretreated patients scored slightly worse in their dysphagia severity index 3 months post-operatively without statistical significance (Fig. 3B).

## DISCUSSION

Treatment options for benign subglottic stenosis include endoscopic as well as open surgical techniques. Although there are no commonly accepted guidelines about the optimal sequence of treatment, patients often receive a dilatation with or without laser enlargement as the primary treatment regime. It has previously been shown by several groups that endoscopic pretreatments do not exclude patients from a definitive surgical repair; however, the impact on voice and swallowing function of such a strategy has never been investigated. To the best of our knowledge, this is the 1st study addressing this important topic.

Functional outcome after laryngotracheal surgery is essential and an important aspect of a shared decision-making. Unfortunately, most surgical series of subglottic stenosis lack a thorough functional assessment before and after surgery. Often, voice quality assessment is limited to a rough categorization into excellent/good voice, impaired voice and no voice without providing any objectifiable measurements. Since the founding of a dedicated airway programme at the Medical University of Vienna, every patient with a subglottic stenosis has received a complete work-up of voice and swallowing [24]. This includes a voice recording of singing and speaking, a quantification of mean fundamental frequency, voice range, dynamic range and sound pressure level of speaking voice as well as subjective assessment tools such as the voice handicap index. This has opened a unique opportunity for internal quality control measures and to determine the impact of surgical repair on voice and swallowing.

It has previously been shown that CTR leads to a significant reduction of the mean fundamental voice frequency due to a loss of cricothyroid joint function. In case of less-extensive disease, technical adaptation of a standard CTR can be feasible in order to preserve the cricothyroid joint. We have previously shown that such a voice-sparing CTR leads to an almost unchanged postoperative voice quality [16]. In the current study, we could show that a voice-sparing approach is less likely in patients with multiple pretreatments. This comes at no surprise, as failed endoscopic treatments usually result in more profound scarring compared to the initial presentation. A more cephalad extension of the scar is commonly observed due to additional damage caused by mechanical trauma/irritation or thermic tissue injury caused by laser. Unlike in treatment-naïve patients, where the planes around the airway are usually well preserved, pre-treated patients often have profound scaring outside the airway, complicating the dissection and mobilization. Malacia, which can develop after multiple failed endoscopic treatment attempts in tracheal stenosis, is not typically seen in the subglottic region.

The preoperative measurements of this series also shows that endoscopic pretreatments impact voice quality *per se*. Pretreated patients had a trend towards a lower mean fundamental frequency (voice pitch) and a lower sound pressure level compared to treatment-naïve patients.

Despite the need for more extended procedures such as an additional partial anterior laryngeal split or lateral cricoplasty, resection lengths, operation times and complication rates were comparable between the 2 study groups. Furthermore, the need for postoperative surveillance at an intensive care unit, start of oral intake and total length of stay was comparable between pretreated and treatment-naïve patients. However, pretreated patients were more likely to develop temporary vocal fold oedema after the procedure. All these facts corroborate the technical feasibility of an open surgical repair after failed endoscopic pretreatments. However, this technical feasibility comes at a certain cost in regard to laryngeal functions.

Several endoscopic techniques have been described in the treatment of benign subglottic stenosis including dilatation with a rigid bronchoscope, bougies, balloon dilatation and the combination of a laser debridement and dilatation. Observational and retrospective studies suggest that endoscopic laser resection may be more effective than dilation; however, there are no prospective studies available comparing both treatments [25, 26]. In addition, only limited evidence is available on the impact on voice quality of different endoscopic treatment modalities [6]. The cohort of this study is too small to account for different techniques of endoscopic pretreatments. However, an important factor of postoperative voice quality was the number of (failed) endoscopic treatment attempts. One or 2 endoscopic treatment trials seemed to affect postoperative laryngeal functions only in a mild way, whereas a clinically significant and persistent impact on voice quality was found in patients who received 3 or more endoscopic treatments.

Endoscopic treatments for benign subglottic stenosis are associated with a high rate of restenosis. Currently, the largest published series comes from the North American Airway Collaboration [27]. This is an ENT-led approach to connect physicians and surgeons with the aim to develop and exchange information concerning the treatment of adult airway disease. More than 75% of patients required a recurrent surgical procedure within 5 years after an endoscopic procedure [6, 7]. This is opposed by excellent long-term results of CTRs [28, 29]. None of the patients included in this study required a reintervention with a median follow-up of 55 months.

### Limitations

This study has several limitations. First, it is a retrospective study and therefore prone to miscoded data or missing values. Since, we prospectively feed every single patients treated in our institution into an airway database, this risk is however minimal. Furthermore, we have no information about voice and swallowing before the endoscopic repair, which would be interesting to know in order to grade the impact of (repeated) endoscopic treatments on laryngeal functions. We acknowledge that this is an institutional experience of a single-centre. Therefore, it might be difficult to generalize our findings. Currently, a dedicated airway section with the ESTS database collects data. We hope to be able to provide multi-institutional data on functional outcome after (crico-)tracheal resections in the near future. Finally, the conclusions from this study are limited by the number of included patients. Despite the relatively high number of laryngotracheal resections performed in our institution, airway procedures are rare in thoracic surgery. Thus, statistical tests have only limited power and some comparisons failed to reach a level of statistical significance. In addition, with such a limited sample size, we cannot exclude confounding factors influencing voice outcomes. Naturally, statistical methods to correct for such factors (multivariate analysis, propensity score matching) cannot be performed.

## CONCLUSION

In conclusion, this work could show that endoscopically pretreated patients require more extended surgical procedures for a definitive repair of a benign subglottic stenosis. In addition, pretreatments are associated with lower voice quality after CTRs.

## Data Availability

The data underlying this article will be shared on reasonable request to the corresponding author. **Matthias Evermann:** Data curation; Methodology; Writing—review and editing. **Thomas Schweiger:** Data curation; Writing—review and editing. **Veronika Kranebitter:** Data curation; Writing—review and editing. **Imme Roesner:** Data curation; Writing—review and editing. **Clemens Aigner:** Writing—review and editing. **Doris-Maria Denk-Linnert:** Data curation; Resources; Supervision; Writing—review and editing. **Konrad Hoetzenecker:** Conceptualization; Formal analysis; Investigation; Resources; Writing—original draft; Writing—review and editing. European Journal of Cardio-Thoracic Surgery thanks Toru Bando, Lucio Cagini, Mohsen Ibrahim and the other, anonymous reviewers for their contribution to the peer review process of this article.

## ABBREVIATIONS

CTR: Cricotracheal resection
ELS: European Laryngological Society
FEES: Flexible endoscopic evaluation of swallowing
GERD: Gastroesophageal reflux disease
GPA: Granulomatosis with polyangiitis
